# Robot‐Assisted Partial Nephrectomy for Multiple Synchronous Renal Tumors in Unilateral Kidney Using Hinotori Surgical System: A Case Report

**DOI:** 10.1002/iju5.70070

**Published:** 2025-07-15

**Authors:** Kento Ozawa, Hiromitsu Watanabe, Kyohei Watanabe, Yuto Matsushita, Keita Tamura, Daisuke Motoyama, Atsushi Otsuka, Teruo Inamoto, Hideaki Miyake

**Affiliations:** ^1^ Department of Urology Seirei Mikatahara General Hospital Hamamatsu Japan; ^2^ Department of Urology Hamamatsu University School of Medicine Hamamatsu Japan; ^3^ Department of Developed Studies for Advanced Robotic Surgery Hamamatsu University School of Medicine Hamamatsu Japan; ^4^ Department of Urology Kobe University Graduate School of Medicine Kobe Japan

**Keywords:** hinotori, multiple renal tumors, robot‐assisted partial nephrectomy

## Abstract

**Introduction:**

Multiple synchronous renal tumors (MSRT) in unilateral kidney are clinically rare. Simultaneous resection for multiple tumors with RAPN is complicated and challenging. Herein, we report the successful resection of three synchronous renal tumors located in unilateral kidney with RAPN using the hinotori surgical robot system.

**Case Presentation:**

A 75‐year‐old man was diagnosed with multiple left renal tumors (45 mm and 20 mm in diameter) that presented very close to each other. We performed RAPN using hinotori and excised the tumors simultaneously. Postoperative renal function was maintained. No complications were identified. The three histological types were distinct: ccRCC, pRCC, and small renal angiomyolipoma.

**Conclusion:**

To the best of our knowledge, this is the first report of MSRT in unilateral kidney successfully treated with RAPN using hinotori.

**Abbreviations**
3D‐CT: three‐dimensional computed tomography
BMI: body mass index
ccRCC: clear‐cell RCC
CECT: contrast‐enhanced computed tomography
eGFR: estimated glomerular filtration rate
MSRT: multiple synchronous renal tumors
POD: postoperative day
pRCC: papillary RCC
RAPN: robot‐assisted partial nephrectomy
RARN: robot‐assisted radical nephrectomy
RCC: renal cell carcinoma
WIT: warm ischemic time

**Summary**

- This is the first report of MSRT in unilateral kidney successfully treated with RAPN using hinotori.
- This case had a good clinical course, suggesting the usefulness of hinotori.

## 1 Introduction

RAPN is widely utilized for small renal masses [1]. Several studies of RAPN for MSRT in unilateral synchronous kidney have been reported; however, its use remains controversial [2]. The da Vinci surgical system (Intuitive Surgical Inc., Sunnyvale, CA, USA) is commonly used for RAPN. To the best of our knowledge, there have been no previous reports of the use of other novel platforms. The hinotori, launched in 2019, is produced by Medicaroid Corporation (Kobe, Hyogo, Japan) and jointly funded by Kawasaki Heavy Industries Ltd. (Kobe, Hyogo, Japan) and Sysmex Corporation (Kobe, Hyogo, Japan), which has unique features [3]. In addition, with the widespread adoption of minimally invasive surgery, the indications for robotic surgery are expanding to include frail and elderly patients, leading to a high demand for new surgical robotic systems [4]. Herein, we describe our initial experience with RAPN using hinotori for MSRT in unilateral kidney.

## 2 Case Presentation

**FIGURE 1:**
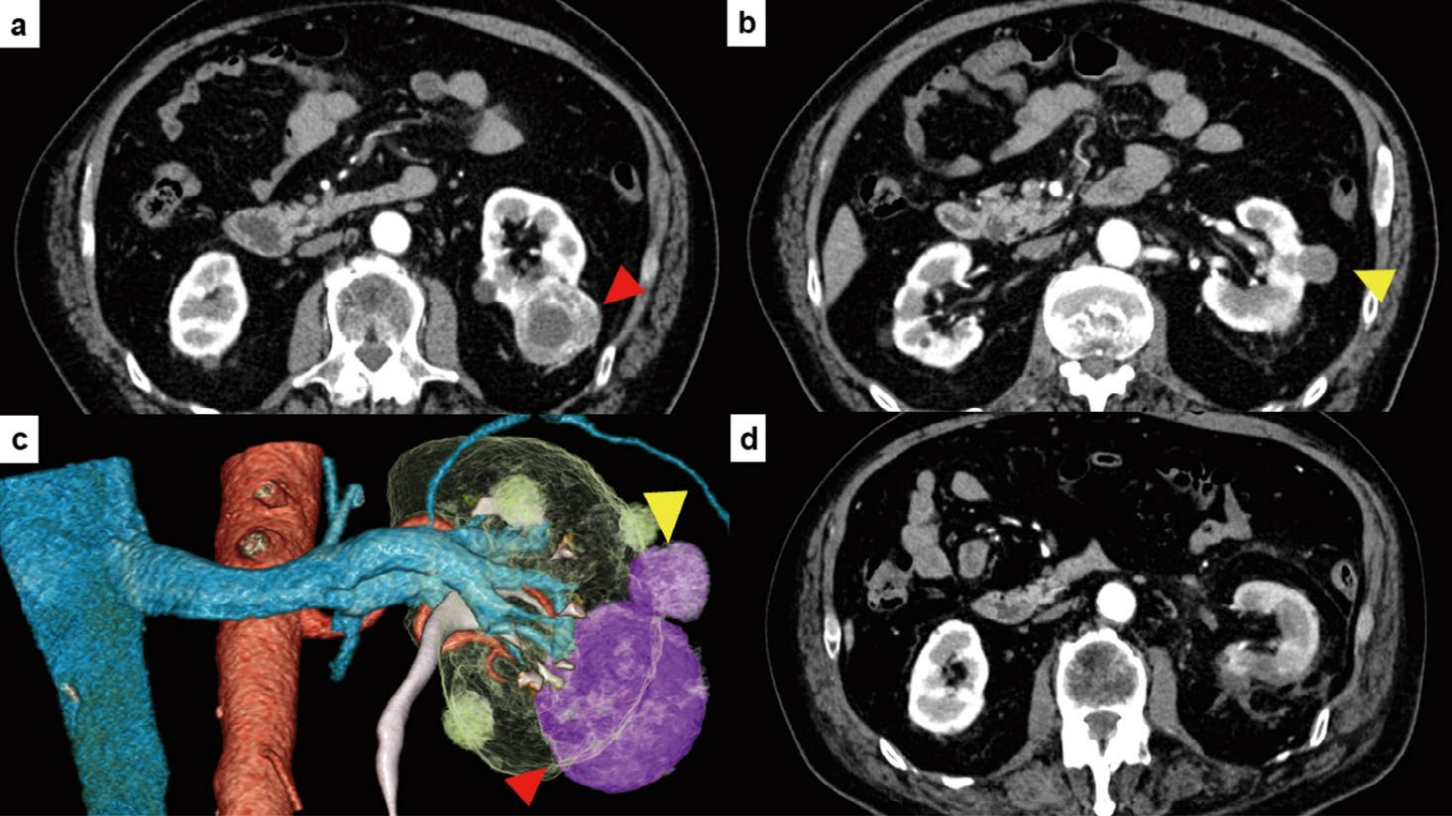
(a, b) Coronal section of contrast‐enhanced computed tomography (CT) of the tumor. (a) Arrows indicate tumors. cT1b tumors showed early staining in the early arterial phase. (b) The cT1a tumor showed gradual staining from the early arterial phase. (c) Three‐dimensional reconstructed image from CT. View from the ventral and slightly caudal side of the left kidney; arrows indicate the tumor. Each tumor was located in close proximity to the other. (d) CT taken on POD4. No pseudoaneurysm or urinary fistula was observed.

A 75‐year‐old male was referred to our institute for further examination of multiple left renal tumors that were incidentally detected. CECT showed two tumors in the lateral mid‐pole of the left kidney: a cT1b tumor and a cT1a tumor (45 mm and 20 mm in diameter, respectively) (Figure 1a,b); R.E.N.A.L. nephrometry scores were 7 and 6 points, with no evidence of metastasis, respectively. 3D‐CT (FUJIFILM Medical Co., Tokyo, Japan) showed that the tumors were located very close together (Figure 1c). Because the patient had multiple comorbidities, including chronic kidney disease (Table 1), we considered RAPN or RARN for curative treatment. Despite the increased surgical difficulty, RAPN was scheduled to prevent worsening renal function. We initiated the procedure after obtaining approval from the Research Ethics Committee of our hospital (Certificate number: 21–091). Written informed consent to receive RAPN was obtained from the patient.

**TABLE 1:** Baseline characteristics.

Age (years)	75
Gender	Male
BMI (kg/m^2^)	30
eGFR (mL/min/1.73 m^2^)	55
CKD stage	3a
Comorbidities	Obesity, hyperlipidemia, and hypertension

Abbreviations: BMI, body mass index; CKD, chronic kidney disease; eGFR, estimated glomerular filtration rate.

**FIGURE 2:**
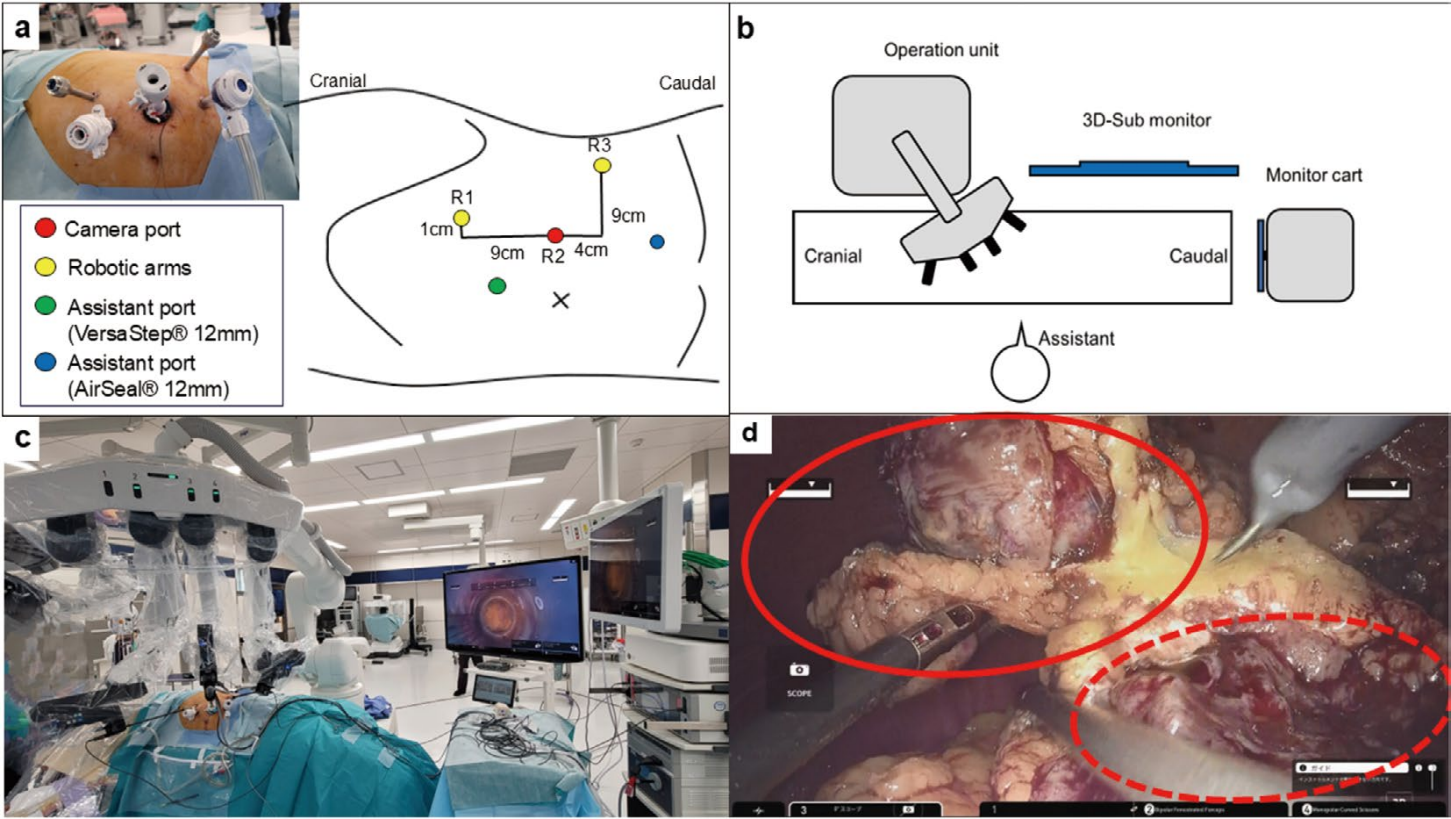
(a) Port positions for left‐side robot‐assisted partial nephrectomy using hinotori. R1‐3: Robot port 1–3. R1, an 8‐mm robotic port for the left arm; R2, a 12‐mm camera port; R3, an 8‐mm robotic port for the right arm; Assistant port, a 12‐mm assistant port. (b) Layout of the surgical instruments. The operation unit is placed on the patient's dorsal and slightly cranial side. We use a 3D‐sub monitor as well as a monitor cart, which are placed on the dorsal and caudal side. An assistant‐surgeon stands on the ventral side. (c) A view of the actual operating room setting for left side RAPN. (d) The two tumors were resected simultaneously (red circle). There was no obvious renal calyx exposure in the resected section (dotted red circle).

**FIGURE 3:**
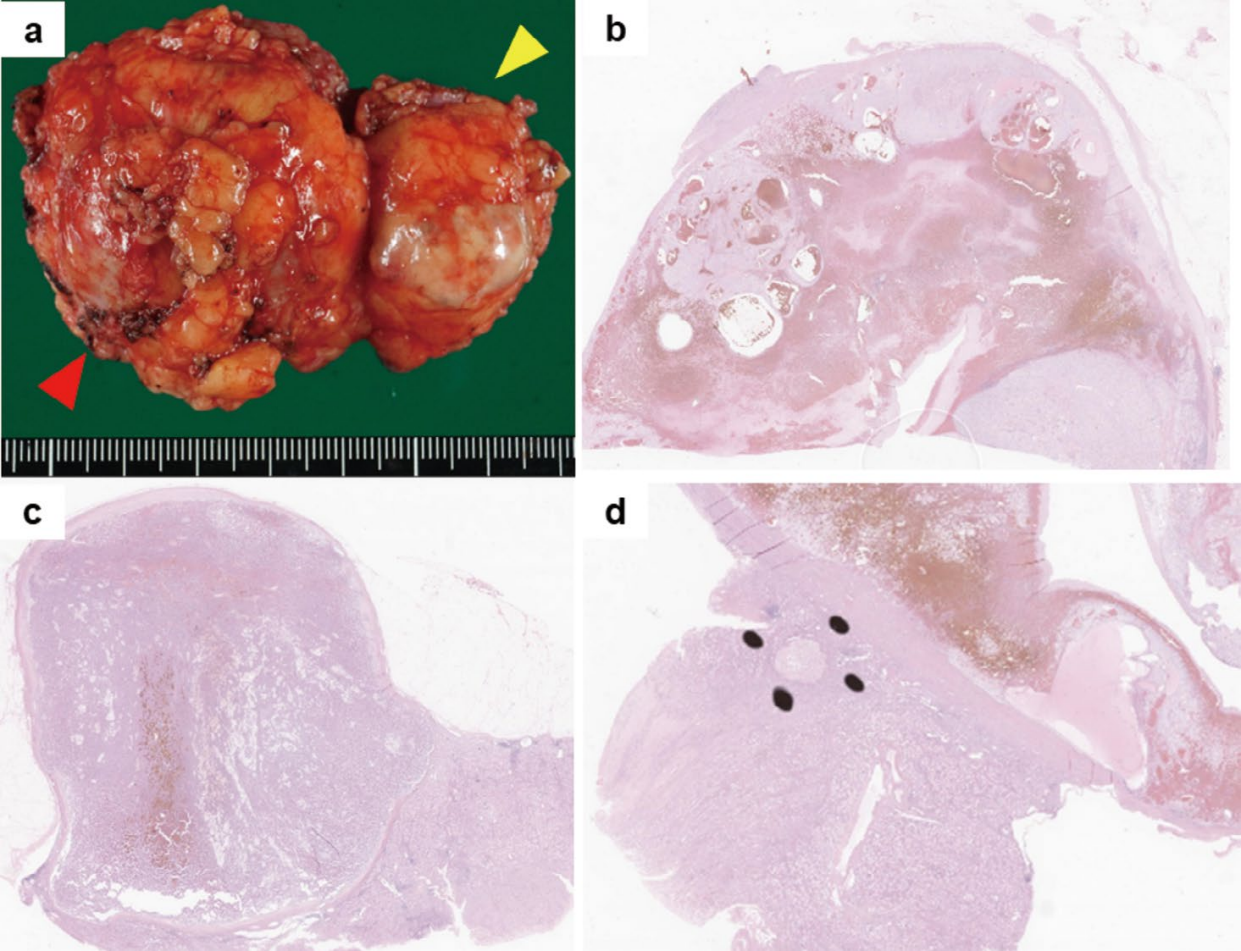
(a) The specimen extracted in this case. The red arrow: The cT1b tumor, which was a yellowish‐enriched mass with hemorrhage and cystic changes. The yellow arrow: The cT1a tumor was a well‐defined, light‐brown, substantial mass. Histological findings of hematoxylin and eosin staining specimens of the three tumors (b–d). (b) ccRCC, pT1a. Tumor cells with pale abundant cytoplasm and nucleoli with distinct enlarged nuclei are seen. Minor venous infiltration is detected. (c) pRCC, pT1a. Tumor cells with eosinophilic cytoplasm and round nuclei are growing in papillary structures with fibrovascular stroma as the axis. (d) Renal angiomyolipoma. A 1.5 mm diameter micronodule composed of spindle‐shaped cells and adipocytes.

The patient's position and placement of trocars were previously reported [5]. Port placement and operation instruments placement are shown in Figure 2. We employed the transperitoneal approach. After the robotic system was mounted, the spleen and descending colon were medially reflected for exposure and securing of the renal artery. After clamping the main renal artery with a Bulldog clamp, the tumors were simultaneously resected using cold scissors, followed by an inner running suture to repair the large vessels (Figure 2d). After the early unclamping of the renal artery, soft coagulation was then added to control the bleeding, followed by renorrhaphy. There was no exposure of the renal calyces. The tumors were completely resected and collected in a specimen bag. The operative outcomes of the present case are shown in Table 2. No pseudoaneurysm or urinary fistula was observed on postoperative CECT (Figure 1d). The patient was discharged on POD 6. The excised specimen and the initial pathologic findings are shown in Figure 3. The cT1b tumor was classified as ccRCC (Figure 3b), and the cT1a tumor was identified as Type 1 pRCC (Figure 3c). Moreover, a small‐diameter renal angiomyolipoma was observed in the excised specimen (Figure 3d). All tumors exhibited negative surgical margins, and trifecta outcomes were achieved. Postoperative 12‐month eGFR was 46, a decrease of 16%.

**TABLE 2:** Comparison with RAPN with multiple synchronous renal tumors in our institute (Reference [9], three cases using da Vinci Xi).

Variables	Case 1	Case 2	Case 3	This case
Type of robotic system	da Vinci	da Vinci	da Vinci	Hinotori
Surgical approach	Trans peritoneal	Trans peritoneal	Trans peritoneal	Trans peritoneal
Time using robotic system (minutes)	109	150	107	162
Warm ischemia time (minutes)	14	15	15	18
Estimate blood loss (mL)	100	5	10	100
Complication	None	None	None	None
Positive surgical margin	Negative	Negative	Negative	Negative
Postoperative eGFR (% of decrease) (1 month after surgery)	31 (29.5)	35 (31.4)	38 (34.5)	36 (34.5)

Abbreviation: eGFR, estimated glomerular filtration rate.

## 3 Discussion

Primary renal malignancies account for about 2%–3% of all malignancies, with most being solitary tumors [6, 7]. The incidence of multiple tumors of different histologic types within a unilateral synchronous kidney is exceedingly rare (0.53%) [8]. The concordance rates are as follows: benign versus malignant 48.6%–91.6%, histological type 58.8%–67.3%, and malignant grade 51.5%–62.5% [6, 9]. Our case was clinically very rare in that ccRCC, pRCC, and a benign lesion (renal angiomyolipoma) were simultaneously present in a unilateral kidney.

RAPN using da Vinci is becoming the standard treatment, with increasing studies reporting its feasibility and safety [1]. A previous study reported its effectiveness for MSRT [2]. We also previously reported three cases of RAPN for MSRT in a solitary kidney using da Vinci [10]. The perioperative outcome of the present case was similar to those of the previous studies (Table 2). Thus, avoiding multiple excisions by excising multiple tumors simultaneously may decrease the risk of perioperative complications [10]. The hinotori is a Japanese‐designed surgical robot system model with several features that differentiate it from da Vinci (Table 3), and frequent software updates are being released that aim to improve the feeling of control. Furthermore, hinotori may be more cost‐effective for the treatment of RAPN. We are actively performing robot‐assisted surgery using hinotori. We previously reported no difference in perioperative outcomes and complications compared with RAPN using da Vinci [11] and reported that 15 patients (50%) had complex renal tumors in our initial series of RAPN using hinotori [5]. Due to the docking‐free design, the arms can readily access the laparoscopic device under the assistant surgeon's control (Table 3). The space around the patient allows the assistant surgeon to work efficiently, which may be helpful for RAPN for high‐complexity tumors. Thus, our findings support previous studies suggesting that RAPN using hinotori is acceptable for treating high‐complexity tumors.

**TABLE 3:** Subjective and objective differences between the two surgical robot systems.

	da Vinci	hinotori
Motion/feeling	Sophisticated and sharper	Straightforward and human‐like manner
Vision	Full high vision	4K, ergonomic design of surgeon cockpit
Operating arms	7‐axis joints, 4 arms, and docking the port to arm.	8‐axis joints, combined with computer control, minimize interference between the arms. 4 arms, and free‐docking due to computer calibrated (Ensures a sufficient working area and reduces external interference with the assistant surgeon)
Cost (RAPN: surgical device)	$ 1245	$ 962

Abbreviation: RAPN, robot‐assisted partial nephrectomy.

RAPN for MSRT in unilateral kidney remains controversial. Because of the increased surgical difficulty compared with that for solitary tumor, it should be performed by a well‐experienced surgeon at a high‐volume center of RAPN; the perioperative complications increase in a tumor‐dose‐dependent manner; and resection of multiple tumors in one lump may increase the amount of intact normal renal tissue removed [12].

Postoperative recurrence in the contralateral kidney is five times more likely in cases of multiple renal tumors compared with cases of solitary tumors [13, 14]. Thus, RAPN for MSRT may be advantageous for preserving renal function. The surgical plan must consider the patient's comorbidities, tumor size, and location (with preparation of 3D‐CT imaging), and the institute's level of proficiency. Moreover, the early unclamping method can be used to shorten the WIT and reduce the risk of pseudoaneurysm [15].

In the present case, because the tumors were very close to each other and the patient had a high risk of future renal function disorder, we resected both tumors simultaneously to preserve renal function. Despite the limitation of being a single‐case experience, this is the first case of RAPN using hinotori for MSRT in a unilateral kidney with a good clinical course, suggesting the usefulness of hinotori.

## Ethics Statement

Approval of the research protocol by an Institutional Review Board, 21‐091.

## Consent

The authors have nothing to report.

## Conflicts of Interest

Miyake Hideaki is an Editorial Board member of the International Journal of Urology and a coauthor of this article. To minimize bias, he was excluded from all editorial decision‐making related to the acceptance of this article for publication. All other authors have no conflicts of interest to declare and received no financial support.
